# COVID-19 incidence in women of reproductive age: a population-based study in Reggio Emilia, northern Italy

**DOI:** 10.1186/s12884-023-06044-z

**Published:** 2023-10-13

**Authors:** Filomena Giulia Sileo, Laura Bonvicini, Pamela Mancuso, Massimo Vicentini, Lorenzo Aguzzoli, Asma Khalil, Paolo Giorgi Rossi

**Affiliations:** 1 Department of Biomedical, Metabolic and Neural Sciences, International Doctorate School in Clinical and Experimental Medicine, University of Modena and Reggio Emilia, Modena, Via Campi 80, 41125 Modena, Italy; 2 Prenatal Medicine Unit, Obstetrics and Gynecology Unit, Department of Medical and Surgical Sciences for Mother, Child and Adult, University of Modena and Reggio Emilia, Via del Pozzo 71, 41125 Modena, Italy; 3 Epidemiology Unit, Azienda Unità Sanitaria Locale -IRCCS Di Reggio Emilia, Via Giovanni Amendola 2, 42123 Reggio Emilia, Italy; 4 Unit of Obstetrics and Gynecology, Azienda Unità Sanitaria Locale—IRCCS, Viale Risorgimento, 80, 42123 Reggio Emilia, Italy; 5 Fetal Medicine Unit, St George’s Hospital, St George’s University of London, Blackshaw Road, TootingLondon, SW17 0QT UK; 6 Vascular Biology Research Centre, Molecular and Clinical Sciences Research Institute, St George’s University of London, Blackshaw Road, Tooting, London, SW17 0QT UK

**Keywords:** SARS-CoV-2 test, COVID-19, Pregnancy, Puerperium, Risk of infection, Incidence

## Abstract

**Background:**

Despite being at higher risk of severe disease and pregnancy complications, evidence on susceptibility to SARS-CoV-2 infection in pregnancy is still limited. The aim of the study is to compare the likelihood of undergoing a SARS-CoV-2 test and testing positive for COVID-19 in pregnancy and puerperium with that of the general female population of reproductive age.

**Methods:**

This is a retrospective population-based cohort study including 117,606 women of reproductive age (March 2020-September 2021) with 6608 (5.6%) women having ≥ 1 pregnancy.

Women were linked to the pregnancy registry to be classified as “non-pregnant”, “pregnant”, and “puerperium”; then, according to the national case-based integrated COVID-19 surveillance system, all women undergoing a SARS-CoV-2 test during the study period were identified. The Incidence Rate Ratio was calculated to compare the likelihood of being tested for SARS-CoV-2 in pregnant, puerperium and non-pregnant women among all women included. The likelihood of having a COVID-19 diagnosis was calculated using two comparators (not-pregnant women and the person-time before/after pregnancy) by means of Cox proportional hazards models, adjusting for age and with the cluster option to control standard error calculation in repeated pregnancies. Only first infection and swabs before the first one positive were included.

**Results:**

The probability of being tested for SARS-CoV-2 was 4.9 (95% CI: 4.8–5.1) and 3.6 times higher (95%CI: 3.4–3.9) in pregnancy (including spontaneous miscarriages) and in the puerperium, respectively.

The Hazard Ratio (HR) of covid-19 diagnosis during pregnancy vs. non-pregnancy was 1.17 (95% CI 1.03–1.33) with similar results when comparing the risk during pregnancy with that of the same women outside pregnancy (puerperium excluded), with an HR of 1.13 (95% CI 0.96–1.33); the excess decreased when excluding the test performed at admission for delivery (HR 1.08 (95%CI 0.90–1.30). In the puerperium, the HR was 0.62 (95% CI 0.41–0.92) comparing women with ≥ 1childbirth with all other women and excluding the first two weeks of puerperium.

**Conclusions:**

Women during pregnancy showed a small increase in the risk of infection, compatible with a higher likelihood of being tested. A lower probability of infection during the puerperium was observed during the entire pandemic period, suggesting likely protective behaviors which were effective in reducing their probability of infection.

## Background

COVID-19, caused by SARS-CoV-2, has spread rapidly around the world since it was declared a pandemic on the 11^th^March 2020 by the World Health Organization [1].

As for other viral infections, pregnancy might represent a vulnerable status with an increased risk of morbidity, disease severity and fatality of COVID-19 [2–5].

Several registries and multinational collaborations [6–8] reported women with SARS-CoV-2 infection to be more than twice as likely as women without the infection to have fetal death [9], experience a preterm birth [9–11], and preeclampsia or eclampsia [12].

Clinical or laboratory findings, admission to Intensive Care Unit, and other outcomes were compared in pregnant vs. non-pregnant women diagnosed with SARS-CoV-2 infection mainly in women admitted to hospital for any reason [10]. Only a few studies have tested asymptomatic pregnant women receiving routine obstetric care without comparing the risks of infection with those of the general population [13–15].

The US Centers for Disease Prevention and Control reported updated information on 1 300 938 women of reproductive age (15–44 years) diagnosed with SARS-CoV-2 [16], including 23434 pregnant women (5.7% of all symptomatic women) but without any information on the incidence of COVID-19 in women of reproductive age or comparison with pregnant or postpartum women.

Furthermore, the puerperium has not been extensively evaluated: studies mainly focused on outcomes of neonates born to SARS-CoV-2 positive mothers or on providing clinical guidance on available treatment in the postpartum period [17, 18].

Pregnancy could potentially influence the risk of infection through both biological and, most importantly, behavioural mechanisms. During pregnancy, women are more likely to be tested for screening or mild symptoms; due to scheduled, undeferrable appointments, they have more contacts with healthcare facilities, thus increasing the risk of being exposed to the virus. Furthermore, during pregnancy and the puerperium, women usually reduce their work and social activities, thus probably reducing the likelihood of infection. Moreover, they might also be more careful in applying physical distancing and using Personal Protection Equipment in order to protect the baby.

Biological mechanisms explaining the differences between pregnant and not-pregnant women include the presence of angiotensin-converting enzyme2 (ACE2) in human placentas, which is the main receptor for SARS-CoV-2 [19]. However, despite the high affinity of SARS-CoV-2 for ACE2, epidemiological evidence on susceptibility to SARS-CoV-2 infection in pregnancy is still limited.

Therefore, the aim of this study is to compare the likelihood of undergoing a SARS-CoV-2 test and testing positive for COVID-19 in pregnancy and puerperium with that of the general female population of reproductive age in the Italian province of Reggio Emilia.

## Methods

### Study design, setting, and population

This is a retrospective population-based cohort study using registry data of the Reggio Emilia Province in northern Italy, which has a population of around 532 000 inhabitants. The Local Health Authority, the local public entity of the Italian National Health Service, provides hospital, outpatient, primary and preventive care to the entire population residing in the province [20].

All women between 15 and 49 years of age and resident in Reggio Emilia Province were included in the study and linked to the COVID-19 surveillance system where all tests and their results are registered. In this way, all women within this cohort who had undergone a SARS-CoV-2 test (also more than once) were identified together with the test’s result.

**Fig. 1:**
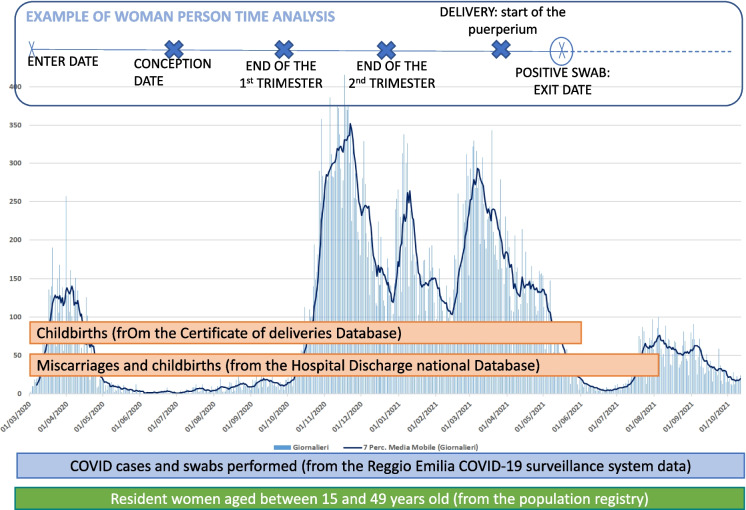
Example of woman’s person-time analysis across the study period. Each woman’s person time starts on the 1^st^ March 2020. If a woman has a pregnancy during the study period, her time from the conception date to the puerperium enters in the pregnancy group. If at any time during the study, a positive swab is registered, then the woman exits the analysis and the following person time is not considered for further analysis

Then, within this cohort, women person-time was classified as “non-pregnant”, “pregnant” or “in puerperium” (within 6 weeks after birth) according to births and/or spontaneous miscarriages occurred from 1^st^ March 2020 up to 30^th^ September 2021 as registered in the Certificate of Delivery Database (CedAP) and the Hospital Discharge Database (SDO) (Fig. 1). In particular, women that were never pregnant in the study period had their person-time only included in “non-pregnant” group while women having a pregnancy in the study period had their person-time classified as “non-pregnant”, “pregnant”, “in puerperium” and again “non-pregnant” after the puerperium.

Women undergoing medical or surgical termination of pregnancy (TOP) during the study period were identified and the corresponding person-time was then excluded from the analysis. For miscarriages, we considered the length of pregnancy to be 3 months.

Furthermore, we decided to include only women till the end of September 2021 to minimize the impact of Omicron variant in our population. In fact, Omicron (B.1.1.529) variant was first identified in November 2021. This variant was characterized by enhanced transmissibility and immune evasion, being able to reinfect individuals previously infected with other SARS-CoV-2 variants and possibly able to evade the immunity in vaccinated individuals [21].

### Data sources

All SARS-CoV-2 tests performed in Italy must be recorded in the national case-based integrated COVID-19 surveillance system [22]. This surveillance system contains data on all COVID-19 patients, collected by the Public Health Department of the Local Health Authority through epidemiologic investigations. We only included PCR tests; antigen self-tests were made available for diagnosis from January 2022 and therefore are not included in this study.

Furthermore, we identified among all SARS-CoV-2 tests recorded, those routinely performed at hospital admission for screening. We could not distinguish between asymptomatic vs. symptomatic women.

The CedAP database is a national registry containing all the epidemiological, socio-demographic and health data regarding the birth event, including data about congenital malformations and perinatal deaths, established by the Italian Ministry of Health in 2001 (Decreto 16 luglio 2001, n° 349). Its use was started in the region of Emilia Romagna in 2002 with additional information in order to implement the regional database; the data from every province in Emilia Romagna are acquired and analyzed every six months [23].

For each hospital admission, all diagnostic information and operative procedures are described according to the modified version of the International Classification of Diseases, ninth revision [24] and Diagnosis Related Groups ver. 24^th ^[25], respectively and recorded into the Hospital Discharge Database (SDO).

**Fig. 2:**
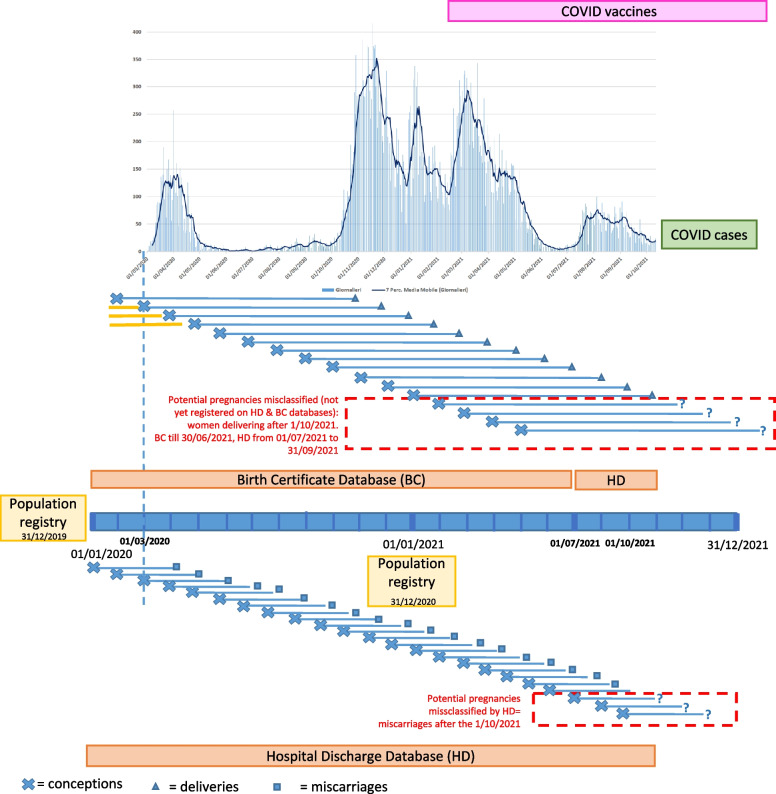
Visual representation of ascertainment bias In the upper part, the number of Covid cases over time In the lower part, woman’s person-time is represented over x-axis from conception date (X) to delivery (Triangle) or miscarriage (Square). In red: potential pregnancies that might be misclassified since delivery or miscarriages occurred after 31^st^ October 2021 (Question mark)

Figure 2 shows how the pregnancy status ascertainment in the study period. Despite the integration of the information with the most timely available data source, SDO, a group of unknown pregnant women remains in the population classified as non-pregnant.

### Main outcomes

From the COVID-19 surveillance system data, all women within this cohort who had undergone a SARS-CoV-2 test were identified and the test result was recorded.

The primary outcome was to calculate the incidence up to 9^th^ November 2021 of being tested for SARS-CoV-2.

The secondary outcomes included the likelihood of receiving a laboratory-confirmed diagnosis of Covid-19, i.e. having a positive test for SARS-CoV-2 (according to the COVID-19 definition adopted by the Italian Ministry of Health, these are all COVID-19 cases) among all women resident in Reggio Emilia Province. Furthermore, we calculated the likelihood of being tested and receiving a laboratory-confirmed diagnosis of Covid-19 according to the trimester of pregnancy and phase of pandemic.

### Statistical analysis

The Incidence Rate (IR) was calculated by taking the total number of swabs and dividing that by the sum of the person-time during which they were made (i.e. “non-pregnant”, “pregnant” and “in puerperium” person-time). The Incidence Rate Ratio (IRR) was calculated to compare the likelihood of being tested for SARS-CoV-2 in pregnancy and in puerperium compared to the likelihood of being tested during non-pregnant person-time. Therefore, the IRR is the ratio between the IR in each category and the IR in non-pregnant women. This ratio was calculated first including all tests and then excluding all swabs routinely performed at admission (for miscarriages) and from 38 weeks onwards to avoid the bias of swabs performed for screening purposes (i.e. at admission for delivery). No other swabs were required for screening purposes by our health system during pregnancy in the study period, but all tests (apart from those at admission) were usually performed in case of symptoms or for contact-tracing.

The likelihood of being tested during pregnancy and during puerperium were compared with that of non-pregnant time; moreover, we also calculated the likelihood of being tested only considering women having at least one childbirth during the study period (always comparing puerperium time and non-pregnant time). We performed a sensitivity analysis by removing the first two weeks from the puerperium in order to remove from the analysis the effect of screening at the time of admission for birth. In the analysis we considered only the swabs performed until the first positive swab (included).

We also calculated the Hazard-Ratio (HR) of covid-19 infection, i.e. the likelihood of receiving a covid-19 diagnosis (only the first infection were considered). This is a non-repeatable outcome that was compared among the three groups, i.e. non-pregnant (reference group), pregnant and in puerperium, by means of Cox proportional hazards models adjusting for age and with the cluster option to control standard error calculation in repeated women (i.e. women having more than one pregnancy).

Analyses were also stratified by three periods defined according to the phases of the pandemic and the restriction measures:

- from 1^st^ March 2020 to 1^st^ June 2020: first phase of the pandemic with spread of wild type variant and lockdown measures in place;
- from 2^nd^ June 2020 to 1^st^ June 2021: period with the spread of Wild type and Alfa variants, various and alternating measures of restriction in place and vaccination not yet widespread among young people;
- from 2^nd^ June 2021 to the end of the study period (9^th^ November 2021): Delta type variant and widespread vaccination among young people.

The analysis was performed using STATA Vers. 16 (StataCorp. 2019. Stata Statistical Software: Release 16. College Station, TX: StataCorp LLC).

### Ethics

The study was approved by the Area Vasta Emilia Nord Ethics Committee (no. 2020/0045199).

Due to the retrospective and observational nature of the data which are routinely collected in daily practice for clinical purposes, the Ethics Committee (Area Vasta Emilia Nord Ethics Committee) authorized the use of patient data, even in the absence of consent, if all reasonable efforts had been made to contact the patient.

All methods were carried out in accordance with declaration of Helsinki.

## Results

We included 117 606 women aged between 15 and 49 years resident in Reggio Emilia province during the study period. Among these, at least one pregnancy occurred in 6608 (5.6%) women (5172 with at least one childbirth, 117 with both childbirth and miscarriages/TOP and 1319 with at least one miscarriage/TOP).

Overall, we observed 5319, 604 and 897 childbirths, spontaneous miscarriages and TOP occurring in the study period, respectively.

**Fig. 3:**
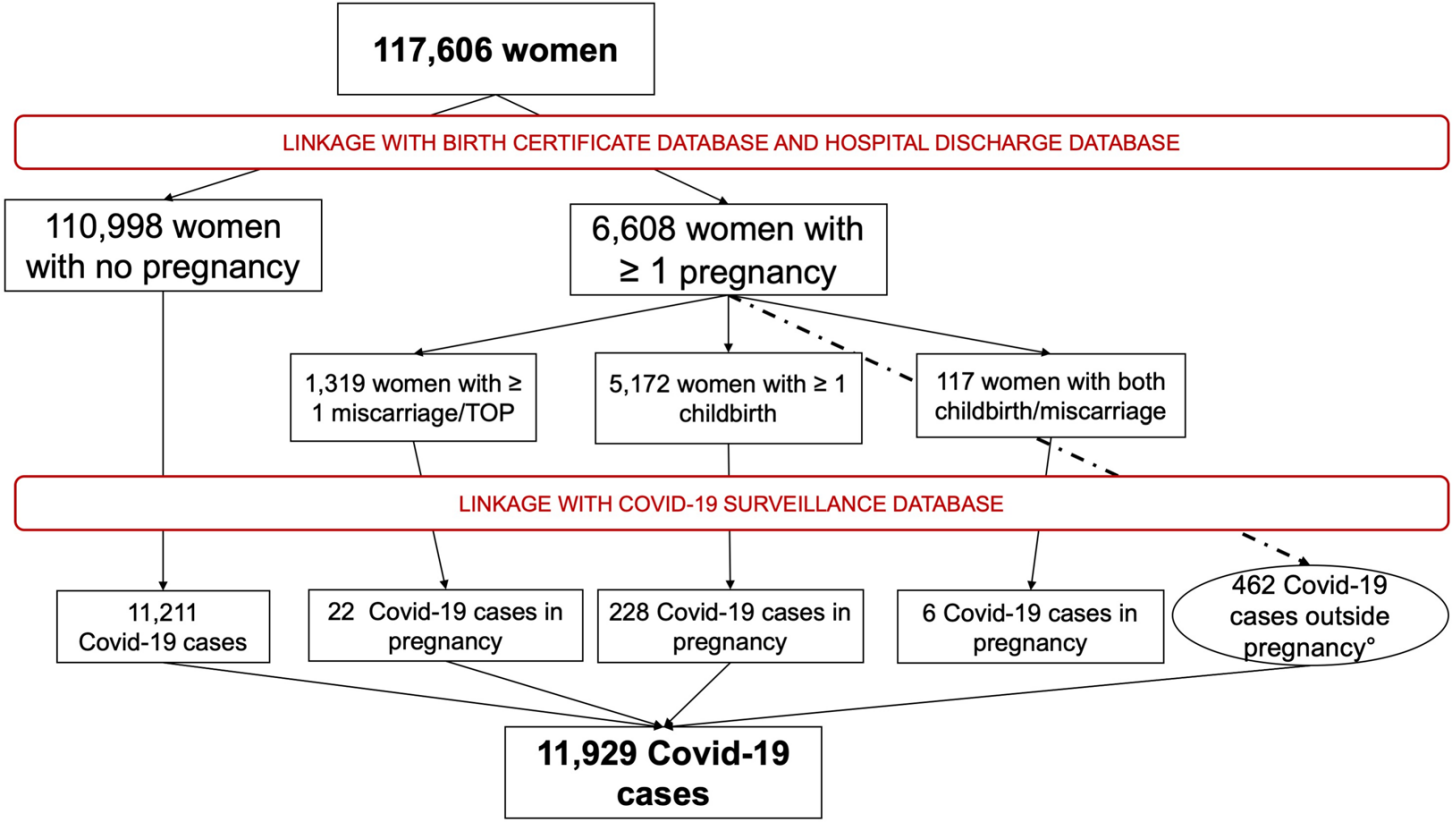
Flow chart of the included women, pregnancies and Covid-19 cases

The total number of COVID-19 cases was 11 929 (10%): among these, 11 211(10%) cases occurred among women that were never pregnant in the study period, 462 (7%) cases occurred among women that had a pregnancy during the study period but they were not positive while they were pregnant (of these cases, 24 occurred during puerperium) and 256 (3.9%) cases of COVID-19 occurred during the pregnancy (Fig. 3).

The total number of swabs performed on these women was 96 945. Table 1 shows for each group (i.e. non-pregnant, pregnant and puerperium) the chances of having a test and, among those tested, the chances of it being positive.

**Table 1:** Chances of having a test and chances of it being positive in the three groups

	Number of women	Number of swabs	**Chances of having a test (%)**	Number of positive swabs	**Chances of having a positive test (%)**
			Number of swabs/ number of women		Number of positive swabs/ number of swabs
Non-pregnant group	110 998	88 326	79.6	11 211	12.7
Pregnant group	5726	7220	126.1	243	3.4
Puerperium group	5319	994	18.7	24	2.4

Total number of included swabs: 96,540 (swabs performed in women having a TOP (*n* = 405) were excluded)

### Likelihood of testing

During the study period, the IR of swabs was 1.3 among all women considering only non-pregnant person-time, 6.4 among those with a pregnancy (IRR of 4.9 (95%CI: 4.8–5.1)) and 4.7 in the puerperium (IRR 3.6 (95%CI: 3.4–3.9)). The IRR for women with a miscarriage was 3.3 (95%CI: 2.9- 3.8) and became significantly lower, i.e. 1.2 (95%CI 0.9–1.5) after excluding all swabs routinely performed at admission. Fifty five percent of women who gave birth had a swab on the day of delivery or within 3 days beforehand.

Considering only pregnancy time till 38 weeks’ gestation in women who gave birth during the study period, the likelihood of having a swab during the third trimester decreased compared to the IRR calculated considering all the pregnancy person-time but remained significantly higher compared to the reference category (IRR: 3.9, 95%CI: 3.7–4.1) (Table 2).

**Table 2:** Swabs risk. Number of swabs, person-time at risk, Incidence Rate (IR) and Incidence Rate ratio (IRR) by state of pregnancy and timing of swab

		**Probability to having a SARS-CoV-2 test**		
	**Person time (days)**	**N.of swabs**	**IR (× 1000)**	**IRR**
**Non pregnant**^**a**^	**67 784 600**	**88 326**	**1.3**	**ref**
All Pregnancies	1 192 627	7625	6.4	4.9 (4.8; 5.0)
**Pregnancies without TOP**	**1 122 487**	**7220**	**6.4**	**4.9 (4.8; 5.1)**
**Spontaneous miscarriages**				
*considering all swabs*	*47 917*	*208*	*4.3*	*3.3 (2.9; 3.8)*
*without swabs at the end of pregnancy*	*47 917*	*73*	*1.5*	*1.2 (0.9; 1.5)*
**Childbirth pregnancies**	1 074 570	7012	6.5	5.0 (4.9; 5.1)
*first trimester*	*281 368*	*336*	*1.2*	*0.9 (0.8;1.0)*
*second trimester*	*358 213*	*400*	*1.1*	*0.9 (0.8;1.0)*
*third trimester*	*434 989*	*6276*	*14.4*	*11.1 (10.8; 11.4)*
*third trimester until the 38th week*	*363 067*	*1858*	*5.1*	*3.9 (3.7; 4.1)*
**Puerperium**	**211 590**	**994**	**4.7**	**3.6 (3.4; 3.9)**

Total number of swabs: 96 945

^a^Puerperium is excluded

### Likelihood of infection

The Hazard Ratio (HR) of covid-19 infection during pregnancy vs. non-pregnancy was 1.17 (95%CI: 1.03–1.33), meaning that the risk of receiving a Covid-19 diagnosis was higher during pregnancy. Results were similar when comparing the risk during pregnancy with that of the same women outside pregnancy and puerperium, with an HR of 1.13 (95%CI: 0.96; 1.33).

In women who gave birth during the study period, no differences in the HR of having a Covid-19 diagnosis in the first trimester or second trimester of pregnancy were found but the HR was increased in the third trimester (Table 3). The increased risk decreased after excluding swabs performed after 38 weeks’ gestation, but remained significantly higher compared to the HR considering all the pregnant person-time (HR: 1.40, 95%CI 1.10; 1.76). The HR was 0.62 (95% CI: 0.41–0.92) comparing the puerperium in women with at least one birth with all other women; and HR was similar when considering only women with a birth during the study period. The HR also remained the same when the first two weeks of the puerperium were excluded from the analysis.

**Table 3:** Probability of receiving a covid-19 diagnosis (HR and 95%CI)

	**Probability of covid-19 diagnosis**		
	*All women*	*Only women with childbirths*	*Only women with childbirths without swabs at the end of pregnancy*^b^
Non-pregnant time^a^ (Ref)	1	1	1
Pregnant time	1.17 (1.03; 1.33)	1.18 (0.99; 1.40)	1.08 (0.90; 1.30)
first trimester		0.82 (0.59; 1.14)	0.85 (0.61; 1.19)
second trimester		0.91 (0.69; 1.19)	0.92 (0.70; 1.21)
third trimester		1.60 (1.30; 1.96)	1.40 (1.10; 1.76)
Puerperium	0.62 (0.41; 0.92)	0.60 (0.40; 0.91)	0.60 (0.40; 0.91)

^a^Puerperium is excluded

^b^without considering person-time and swabs from 38 weeks’ gestation onward

### Different phases of pandemic

Analysis by phase of the pandemic did not show any differences in terms of the risk of having a covid-19 diagnosis. We observed a lower risk in the first and second trimester during the first phase of the pandemic compared to the subsequent period (Table 4).

**Table 4:** Hazard Ratio (HR) of receiving a covid-19 diagnosis by period in women with at least one childbirth

		*01/03/2020—01/06/2020*			*02/06/2020—01/06/2021*			*02/06/2021—9/11/2021*		
	HR	*N*	Person-time	HR	*N*	Person-time	HR	*N*	Person-time	HR
Non-pregnant time^*^	1	19	216 735	1	230	935 281	1	61	656 331	1
Pregnant time	1.18 (0.99; 1.40)			0.90 (0.47; 1.70)			1.21 (1.01; 1.46)			0.90 (0.31—2.60)
first trimester	0.82 (0.59; 1.14)	3	79 797	0.42 (0.12; 1.41)	38	201 542	0.89 (0.63; 1.26)	0	29	-
second trimester	0.91 (0.69; 1.19)	4	83 165	0.55 (0.18; 1.62)	60	271 117	0.95 (0.72; 1.26)	0	3931	-
third trimester	1.60 (1.30; 1.96)	12	75 435	1.81 (0.88; 3.72)	112	302 949	1.63 (1.30; 2.04)	4	56 605	0.91 (0.31; 2.63)
Puerperium	0.60 (0.40; 0.91)	1	26 164	0.43 (0.06; 3.26)	21	137 238	0.65 (0.41; 1.01)	2	48 188	0.42 (0.10; 1.70)

^*^Puerperium is excluded

The number of swabs performed was lower in the first period of the pandemic and increased in the subsequent period. The IRR comparing the likelihood of having a swab in the third trimester with that during non-pregnant time was 1.99 (95%CI:1.48- 2.65) in the first period and 22.84 (95%CI: 20.85- 25.05) in the third period (Table 5).

**Table 5:** Number of swabs, person-time at risk, Incidence Rate (IR) and Incidence Rate Ratio (IRR) by state of pregnancy and time period in women with at least one childbirth

	*01/03/2020—01/06/2020*				*02/06/2020—01/06/2021*				*02/06/2021—9/11/2021*			
	*N*	Person-time	IR	IRR	*N*	Person-time	IR	IRR	*N*	Person-time	IR	IRR
Non-pregnant person-time^*^	120	216 735	0.55	ref	1372	935 281	1.47	ref	703	656 331	1.07	ref
Pregnant person-time												
first trimester	28	79 797	0.35	0.63 (0.40; 0.96)	308	201 542	1.53	1.04 (0.92; 1.18)	0	29	0.00	-
second trimester	21	83 165	0.25	0.46 (0.27; 0.73)	373	271 117	1.38	0.94 (0.83; 1.05)	6	3931	1.53	1.42 (0.52; 3.12)
third trimester	83	75 435	1.10	1.99 (1.48; 2.65)	4808	302 949	15.87	10.82 (10.19; 11.50)	1385	56 605	24.47	22.84 (20.85; 25.05)
Puerperium	26	26 164	0.99	1.79 (1.13; 2.76)	568	137 238	4.14	2.82 (2.55; 3.11)	400	48 188	8.30	7.75 (6.84; 8.77)

^*^Puerperium is excluded

## Discussion

The likelihood of being tested was around 10% lower during the first and second trimesters of pregnancy than in non-pregnant women, while it was 10 times higher in the third trimester. The probability of having a covid-19 diagnosis (independently from the number of swabs) was similar to that of the general population in the first two trimesters but around 60% higher in the third trimester; this excess risk decreased to 40% when excluding the routine swabs taken on admission. Overall, the chance of having a Covid-19 diagnosis (i.e. a positive test) was higher during pregnancy and the excess almost disappeared when we excluded the routine screening swab on admission, despite the probability of being tested during pregnancy still being three times higher.

Similarly, in the puerperium there was a higher probability of being tested but the likelihood of having a Covid-19 diagnosis was lower than in the general population.

These findings were consistent through the different phases of the pandemic. The only appreciable difference was a lower probability of being tested and of infection in the first and second trimesters in the first wave of the pandemic, i.e. during the period of strict lockdown.

To our knowledge, this is the first study comparing the likelihood of being tested and of infection in women of reproductive age according to their pregnancy status. Furthermore, other strengths of the study are the large number of included women and the availability of data from the COVID-19 surveillance system, which allowed to identify all women who had undergone a SARS-CoV-2 test and its result. Moreover, during the study period, the testing strategies were homogeneous: all women at admission were tested for SARS-CoV-2, no tests were required for screening purposes during the pregnancy (for example to have access to routine appointments). In the whole population, tests were usually performed in case of symptoms or for contact-tracing. Home-testing were made available in the end of April 2021 in Italy. However, a positive result still needed external confirmation to allow the diagnosis of covid-19. Therefore, we don’t think this might have underestimated the diagnosis in the study period.

Our study has several limitations. First, there is a risk of ascertainment bias. In particular, there was the risk of misclassification of some women and their status (pregnant but not yet delivered) due to the fact that data about deliveries are available only from six months after the delivery date (Fig. 2). The number of miscarriages might also have been underestimated when occurring spontaneously and not requiring any form of hospitalization.

Another limitation was that we did not know whether the test was performed as a screening or for symptoms, therefore it has been inferred that all those performed for miscarriages or pregnancies of 38 weeks onwards were screening tests. This might have led to consider as a screening test the swab made in a 38-week symptomatic pregnant woman. On the other hand, some screening tests were not excluded correctly, for example, those made for screening in women admitted for preterm labor or maternal complications during pregnancy before term. Therefore, being the information on symptoms not available, the clinical impact of the higher incidence of Covid-19 infection in pregnant women cannot be interpreted.

Furthermore, we did not consider the vaccination status in our population which might have differed considerably between pregnant and non-pregnant women. In particular, by the end of July 2021 the mean first dose coverage among non-pregnant women was around 63%, rising up to 83% by the end of October 2021 [26, 27]. On the other hand, all pregnant women were invited to vaccination against SARS-CoV-2 only from September 2021 (and in the second and third trimester of pregnancy) while before that date the vaccination was strongly advised only for women at high risk of infection or with comorbidities [28].

Finally, the non-pregnant group might also have been overestimated because of migrations or deaths not yet registered. Nevertheless, given the short period of follow-up since the last residence check, i.e. maximum of 12 months, this misclassification should not have produced a strong bias.

Despite the abundant literature on pregnancy and Covid-19, few studies have compared the risk of being tested and of being positive in the general population of women of reproductive age compared with pregnant women. For example, in the large cohort of more than 1 million women of reproductive age with laboratory-confirmed infection with SARS-CoV-2, the pregnancy status was known in only 461 825 women thus limiting the comparison between pregnant and not-pregnant women [16]. That study confirmed previous reports showing an increased risk of severe COVID-19 among pregnant women, but no information on the risk of infection during pregnancy has been reported.

Our results and sensitivity analyses suggest that the excess risk of having a Covid-19 diagnosis is due, in large part, to the greater likelihood of being tested during pregnancy.

The remaining differences are likely reflecting behavioral changes between the two groups.

Several studies have analyzed trends in hospital admissions in gynecological or obstetrical emergency services during the pandemic, showing a decreased rate of admission across the country [29–31]. On the other hand, rates of routine services offered during pregnancy such as first trimester screening tests or invasive prenatal diagnosis showed different trends in different areas [32, 33].

In our cohort, we observed a reduced chance of having a Covid-19 diagnosis in pregnant women in the first two trimesters in the first wave of the pandemic, when there was a strict lockdown in Italy including a complete stop of all non-essential activities, home working for all, closed schools and stop of any leisure indoor and outdoor activity. These interventions were based on the knowledge that transmission of the virus occurs easily in situations where people gather in close proximity, for prolonged periods of time, indoors and without any physical barriers, such as face masks, which act as a form of source control [34]. It is surprising that, despite pregnant women needing to attend clinics for antenatal visits, thus exposing themselves to the risk of infection, they had a reduced risk in the first wave, when the whole population was under strict restrictions. We expected an opposite trend, i.e. pregnant women could be more protected than the general population when the restrictions were relaxed and their greater motivation to stay safe could make the difference. A possible explanation could be that a large proportion of infections diagnosed in the first wave were acquired before lockdown, when the differences in behavior between pregnant and non-pregnant women were still not appreciable. However, it must be considered that the observed differences in pregnant women’s probability of infection during the pandemic phases are compatible with random fluctuations and that in the first phase of the pandemic, screening was practically absent. Indeed, only 3.5% of women who gave birth during this phase had a swab on the day of delivery or during the 3 days beforehand.

Only during the puerperium, we did observe a reduction in the risk of infection that cannot be mainly attributed to a different frequency of testing. The reduced risk of being diagnosed with covid-19 infection in puerperium cannot be only the effect of pre-partum screening; in fact, even excluding the first two weeks of puerperium, the probability of being diagnosed with covid-19 remains lower, with an unchanged HR. A behavioral explanation is plausible since in this period women usually do not work and are likely to reduce their social activities. Nevertheless, the immune system in breastfeeding women has specific changes and a role in preventing infections may not be excluded [35].

## Conclusion

Women during pregnancy were more likely to receive a laboratory-confirmed diagnosis of Covid-19. This excess is probably due to a higher probability of being tested. We also observed a lower probability of infection during the puerperium, throughout the entire pandemic. This difference suggests that women during the puerperium may have had protective behaviors which were effective in reducing their probability of infection.

## Data Availability

The data underlying this article will be shared on reasonable request to the corresponding author.

## Abbreviations

CedAP: Certificate of Delivery Database
HR: Hazard-Ratio
IR: Incidence Rate
IRR: Incidence Rate Ratio
SDO: Hospital Discharge Database
TOP: Termination of Pregnancy
